# Potassium Permanganate as an Antidote to the Venom of Serpents

**Published:** 1882-03-20

**Authors:** M. DeLacerda

**Affiliations:** (Rio Janeiro)—Gazette des Hopitaux


### POTASSIUM PERMANGANATE AS AN ANTIDOTE TO THE VENOM OF SERPENTS.

A NOTE COMMUNICATED TO THE FRENCH ACADEMY BY M. DE LACERDA (RIO JANEIRO)—*Gazette des Hopitaux.*

TRANSLATED BY DR. A. D. MATAS.

In order to study the action of certain chemical and botanical substances upon the phenomena which follow the inoculation of the venom of serpents, we began two months ago a series of experiments, which have led to results of the highest scientific and practical importance.

After recognizing the worthlessness of the perchloride of iron, borax and nitrate of mercury, tannin and various other chemical substances in relieving the effects of poisonous ophidian inoculations, we determined to try a substance which furnished us truly wonderful results—we refer to the permanganate of potassium.

The results obtained in the first series of experiments, by injecting the active venom of the bothrops, diluted in distilled water, into the subcutaneous areolar tissue of dogs, demonstrated plainly that this substance (the permanganate) was capable of arresting completely the local toxic manifestations of the venom. In carrying out our experiments we proceeded thuswise: A sufficient quantity of cotton was subjected to the bites of the reptile, and the venom collected in its meshes was diluted in a small quantity of distilled water (usually 8 to 10 grains of this menstruum); a Pravaz syringe was then filled with this solution and half of its contents injected into the cellular tissue of the buttock of a dog. One or two minutes after, or even after a longer lapse of time, an equal quantity of a filtered 1 per cent. solution of potassium permanganate was injected into the same region. These dogs, when examined the next day, did not present the slightest local lesion, at most a very small tumefaction, localized about the seat of the injection, without any infiltration or any other sign of irritation. And yet, the same venom which had served in these experiments, when injected without the antidote, always produced great local tumefaction and more or less voluminous abscesses, associated with considerable loss of substance and destruction of tissue.

The results achieved in this first series of experiments by the simple hypodermic injection of potassium permanganate encouraged us to try the same substance in cases of poisoning from the intravenous injection of the ophidian venom.

Here, again, the permanganate of potash answered perfectly to our expectations. We have now repeated our experiment 30 times, and have barely met with two failures. These failures, however, may be attributed to various causes, which do not affect the general efficiency of the agent now under discussion. First, we may state that these experiments were conducted, in these exceptional instances, on badly nourished, very feeble and young animals; and again, the injections of the permanganate were administered too long after the introduction of the poison, when the heart's action was on the verge of suspension.

For a certain number of cases we injected intravenously half a syringeful (Pravaz's syringe) of a solution of the strength of 12 or 15 serpent bites (on cotton) in 10 grains of water, followed half a minute later, by 2 centigrams of a 1 per cent. solution of potassium permanganate. Outside of a very transient agitation, and sometimes of an acceleration in the cardiac pulsations, which hardly ever lasted over a few minutes, the animal experimented upon never manifested the slightest morbid phenomena. The inoculated animals were carefully observed for several days after the experiment, but they always seemed to be perfectly healthy.

In another series of cases, we injected the venom into the bladder and waited for the manifestation of the characteristic poisonous symptoms. Just at the moment when the pupils were observed to be greatly dilated, when respiratory and cardiac troubles, contracting involuntary micturition and defecation, indicated a condition of profound intoxication, we injected intravenously, 2 to 3c. centimeters of the same 1 per cent. solution of potassium permanganate, repeating the injections, one after another, until we observed, usually at the end of 2 or 3 minutes, or five at most, that the above phenomena disappeared; a condition of general prostration was the only perceptible result of the experiment, and this was hardly ever protracted over 15 or 25 minutes. If the animal was then placed on his feet, he could walk very well; he could run if compelled to, and in fact deported himself throughout like a healthy dog. And yet, other dogs that had received within their veins the same quantity of pure venom, without any counteracting agent, died every one, more or less rapidly.

These truly remarkable results, which attracted general attention, were witnessed at different times, not only by His Majesty, Don Pedro, who kindly honored us with his presence in our first experiment, but also by persons highly conversant in these matters, physicians, members of faculties, and members of foreign diplomatic corps, etc.

I therefore believe myself justified in asserting that the permanganate of potassium is a true antidote to the venom of serpents.—*New Orleans Med. and Surg. Journal.*

